# Draft Genome Sequence of *Lactobacillus hominis* Strain CRBIP 24.179^T^, Isolated from Human Intestine

**DOI:** 10.1128/genomeA.00662-13

**Published:** 2013-08-22

**Authors:** Sylvie Cousin, Sophie Creno, Laurence Ma, Dominique Clermont, Valentin Loux, Chantal Bizet, Christiane Bouchier

**Affiliations:** Institut Pasteur, Centre de Ressources Biologiques de l’Institut Pasteur, Département de Microbiologie, Paris, Francea; Institut Pasteur, Plate-forme Génomique, Département Génomes et Génétique, Paris, Franceb; INRA, UR1077 Mathématique, Informatique et Génome, Jouy-en-Josas, Francec

## Abstract

We report the draft genome sequence of the strain *Lactobacillus hominis* CRBIP 24.179^T^, isolated from a human clinical sample. The total length of the 28 contigs is about 1.9 Mb, with a G+C content of 37% and 1,983 coding sequences.

## GENOME ANNOUNCEMENT

*Lactobacillus hominis* strain 61D^T^, renamed *L. hominis* CRBIP 24.179^T^, was isolated in the early 1960s from a clinical isolate (1). Some species of the genus *Lactobacillus* have been isolated from clinical patients with a variety of clinical problems (2, 3). Most of those strains (but not all) belong to the species *Lactobacillus rhamnosus, Lactobacillus casei, Lactobacillus paracasei*, and *Lactobacillus plantarum*. These species are also among those most frequently found in human intestinal flora. Nevertheless, there is only limited information concerning the risk that is linked to potential virulence factors present in *Lactobacillus* strains (4).

Here, we report the genome sequence of *L. hominis* CRBIP 24.179^T^, obtained using a whole-genome strategy based on Illumina paired-end sequencing, with an insert length of 380 bp (Illumina genome analyzer HiSeq 2000). In order to test assembly *de novo* tools, quality-filtered reads (116,085,864 reads, 99 bases mean read length, ~5,190-fold coverage) were assembled using two softwares, VelvetOptimiser version 2.2.0 (5) and ABySS version 1.2.6 (6). The resulting contigs were compared using Mauve version 2.3.1 (7). The two scaffolds were somewhat similar (28 Velvet contigs and 37 ABySS contigs, with maximum lengths of 365,062 and 267,206 bases, respectively). Nevertheless, differences related to the number and the maximum length of contigs and Mauve assessment support the *de novo* assembled contigs obtained using Velvet.

The draft genome consists of 36 contigs, with a total length of 1,927,726 nucleotides (nt) and a G+C content of 37%. The sizes of the contigs were between 701 bases for the shortest and 365,062 bases for the longest. The contigs were annotated with the AGMIAL platform (8), an integrated bacterial genome annotation system. The prediction of coding sequences used the self-training gene detection software SHOW based on hidden Markov models (http://genome.jouy.inra.fr/ssb/SHOW/). tRNAs and rRNAs were detected using tRNAscan-SE (9) and RNAmmer (10) softwares, respectively. There were 1,983 coding sequences (CDSs) predicted (1,938 complete), as well as one rRNA operon with 1 copy each of the 23S, 5S, and 16S genes. Fifty-four tRNA genes were also predicted.

### Nucleotide sequence accession numbers. 

The strain is publicly available in two European collections under the no. CRBIP 24.179^T^ and DSM 23910^T^. The draft of this whole-genome sequencing project has been deposited in EMBL under the accession no. CAKE01000001 to CAKE01000036. The version described in this paper is the first version.

## References

[1] Cousin S Motreff L Gulat-Okalla ML Gouyette C Spröer C Schumann P Begaud E Bouchier C Clermont D Bizet C 2013 *Lactobacillus pasteurii* sp. nov. and *Lactobacillus hominis* sp. nov. Int. J. Syst. Evol. Microbiol. 63:53–59 22328611 10.1099/ijs.0.036665-0

[2] Aguirre M Collins MD 1993 Lactic acid bacteria and human clinical infection. J. Appl. Bacteriol. 75:95–107 8407678 10.1111/j.1365-2672.1993.tb02753.x

[3] Bourne KA Beebe JL Lue YA Ellner PD 1978 Bacteriemia due to *Bifidobacterium*, *Eubacterium* or *Lactobacillus*, twenty-one cases and review of the literature. Yale J. Biol. Med. 51:505–512 749356 PMC2595696

[4] Gasser F 1994 Safety of lactic acid bacteria and their occurrence in human clinical infections. Bull. Inst. Pasteur 92:45–67

[5] Zerbino DR Birney E 2008 Velvet: algorithms for *de novo* short read assembly using de Bruijn graphs. Genome Res. 18:821–829 18349386 10.1101/gr.074492.107 PMC2336801

[6] Simpson JT Wong K Jackman SD Schein JE Jones SJ Birol I 2009 ABySS: a parallel assembler for short read sequence data. Genome Res. 19:1117–1123 19251739 10.1101/gr.089532.108 PMC2694472

[7] Darling AC Mau B Blattner FR Perna NT 2004 Mauve: multiple alignment of conserved genomic sequence with rearrangements. Genome Res. 14:1394–1403 15231754 10.1101/gr.2289704 PMC442156

[8] Bryson K Loux V Bossy R Nicolas P Chaillou S van de Guchte M Penaud S Maguin E Hoebeke M Bessières P Gibrat JF 2006 AGMIAL: implementing an annotation strategy for prokaryote genomes as a distributed system. Nucleic Acids Res. 34:3533–3545 16855290 10.1093/nar/gkl471 PMC1524909

[9] Lowe TM Eddy SR 1997 tRNAscan-SE: a program for improved detection of transfer RNA genes in genomic sequence. Nucleic Acids Res. 25:955–964 9023104 10.1093/nar/25.5.955 PMC146525

[10] Lagesen K Hallin P Rødland EA Staerfeldt HH Rognes T Ussery DW 2007 RNAmmer: consistent and rapid annotation of ribosomal RNA genes. Nucleic Acids Res. 35:3100–3108 17452365 10.1093/nar/gkm160 PMC1888812

